# Effects of temperature and grazing on soil organic carbon storage in grasslands along the Eurasian steppe eastern transect

**DOI:** 10.1371/journal.pone.0186980

**Published:** 2017-10-30

**Authors:** Yanyun Zhao, Yong Ding, Xiangyang Hou, Frank Yonghong Li, Wenjun Han, Xiangjun Yun

**Affiliations:** 1 School of Ecology and Environment, Inner Mongolia University/Inner Mongolia Key Laboratory of Grassland Ecology, Hohhot, China; 2 Grassland Research Institute of the Chinese Academy of Agricultural Sciences/ Key Laboratory of Grassland Ecology and Restoration of the Ministry of Agriculture, Hohhot, China; Beijing Normal University, CHINA

## Abstract

Soil represents the largest terrestrial organic carbon pool. To address global climate change, it is essential to explore the soil organic carbon storage patterns and their controlling factors. We investigated the soil organic carbon density (SOCD) in 48 grassland sites along the Eurasian steppe eastern transect (ESET) region, which covers the Inner Mongolia grassland subregion and Mongolia grasslands subregion. Specifically, we analyzed the SOCD in the top 30 cm soil layer and its relationships with climatic variables, soil texture, grazing intensity and community biomass productivity. The results showed that the average SOCD of the ESET was 4.74 kg/m^2^, and the SOCD of the Inner Mongolia grassland subregion (4.11 kg/m^2^) was significantly lower than that of the Mongolia grassland subregion (5.79 kg/m^2^). Significant negative relationships were found between the SOCD and the mean annual temperature (MAT), mean annual precipitation (MAP) and grazing intensity in the ESET region. The MAT and grazing intensity were identified as the major factors influencing the SOCD in the ESET region; the MAP and MAT were the major factors influencing the SOCD in the Inner Mongolia grassland subregion; and the MAT and soil pH were the major factors influencing the SOCD in the Mongolia grassland subregion.

## Introduction

Soil is the largest terrestrial organic carbon pool and stores two-thirds of the total terrestrial organic carbon. Moreover, soil contains three times as much carbon as the vegetation organic carbon pool and twice as much carbon as the atmosphere [1]. Small changes in the soil organic carbon pool have a significant impact on the global carbon balance, which in turn affects global climate change [2]. Thus, exploration of organic carbon storage in soil and the factors that control this process would be significant in research on the global terrestrial carbon cycle and for carbon budget management [3,4,5]. In recent decades, greater attention has been given to soil organic carbon storage under various scenarios of climate and land use change [6,7,8].

A series of studies have shown that the environmental factors controlling soil organic carbon differ depending on the scale [5,9,10]. At the global and regional scales, temperature and precipitation are dominant factors affecting soil organic carbon storage, and the soil organic carbon increases with increasing precipitation and decreasing temperature [11,12,13]. At the subregional scale, in addition to temperature and precipitation, soil texture plays an important role in soil organic carbon storage [4,9]. For example, the soil clay content has a significant positive effect on soil organic carbon storage [4,14], and soil organic carbon storage increases with an increasing soil pH [9,15]. At the local scale, topography usually has the greatest indirect influence on soil organic carbon storage by affecting the redistribution of temperature and water [10,16]. The vertical distribution of soil organic carbon storage is also controlled by a range of environmental factors: soil organic carbon storage in topsoil is mainly determined by climatic variables, whereas that in subsoil shows a significant association with soil texture [7,13,17]. The soil organic carbon content depends on the balance between the carbon input and output, and environmental factors affect soil organic carbon by impacting the input and/or output of carbon [6,18]. Regarding soil organic carbon storage in terrestrial ecosystems, the carbon input is related to plant productivity, whereas the carbon output mainly depends on microbial organic matter decomposition [5,19]. Generally, under an arid climate, areas with hotter temperatures show lower annual biomass productivity and higher microbial organic matter decomposition than those with cooler temperatures [7,20]. Both plant productivity and microbial organic matter decomposition increase with increasing precipitation, but the relative increases in plant productivity are greater [13,17]. In addition to climate, soil texture plays an important role [9,21]. With an increasing soil clay content, the fixation of soil organic matter is enhanced, and the microbial organic matter decomposition of soil organic carbon decreases, ultimately leading to a higher soil organic carbon content [13,22]. A soil pH that is too high or too low also decreases microbial organic matter decomposition, thereby increasing soil organic carbon storage [9].

Land use change (transformation from one ecosystem to another ecosystem) is also generally considered a major factor controlling soil organic carbon storage [2,7,23,24]. During the ecosystem transformation process, disruption of the carbon input and output is inevitable, and this disruption results in changes in carbon sources and sinks [23]. A variety of land use patterns, such as deforestation [24], agricultural reclamation [7], grazing [25,26], plantation forests [27] and fertilization [4], affect soil organic carbon storage. Many studies have demonstrated that soil organic carbon storage decreases with a shift from a natural land use pattern to an artificial land use pattern, and conversely, soil organic carbon storage increases with a shift from an artificial land use pattern to a natural land use pattern [6,23]. Lal [2] found that land use changes have resulted in a decrease in the cumulative soil organic carbon content by approximately 55–78 Gt since 1750, and it is hoped that 50–60% of the loss of soil organic carbon can be ameliorated through a series of restoration management measures.

Grasslands are an important component of the terrestrial ecosystem, accounting for 26% of its total area and playing a crucial role in the global carbon cycle [28]. Grazing is the most dominant land use practice in grasslands, with 54% of grasslands having been converted to managed grazing lands [29]. With an increasing grazing intensity, a number of different patterns of effects in soil organic carbon have been found, and these include positive, negative and unimodal effects, as well as an absence of a significant relationship [19,30,31]. Grazing not only affects the carbon input by altering plant productivity but also can change the soil clay content and soil pH, which affect microbial organic matter decomposition [15,19,32].The steppe of the Mongolian plateau is located in the eastern part of the Eurasian steppe, which is the largest grassland in the world, stretching over 8000 km from northeastern China, through Inner Mongolia, Mongolia, Russia, and Ukraine, to Hungary [33]. Moreover, next to the Arctic and the Qinghai-Tibet Plateau, the Mongolia plateau is a region that is highly sensitive to global climate change [34]. The Mongolia plateau is divided into two administrative regions, Inner Mongolia and Mongolia. Several ecosystem types are observed from east to west in Inner Mongolia and Mongolia, and these follow a trend of decreasing precipitation from forests to grasslands to deserts. Grasslands occupy approximately 50% of the area in each region. However, since the political separation of Inner Mongolia and Mongolia in 1921, the two regions have developed significant population, socioeconomic, land use and grazing intensity differences [35]. A number of studies have investigated soil organic carbon storage and controlling factors in the Inner Mongolia grassland subregion or Mongolia grassland subregion, but to the best of our knowledge, the difference in soil organic carbon storage between these two regions has not been investigated [21,36,37,38].

To better understand the effects of climatic variables, soil texture, vegetation and grazing intensity on soil organic carbon storage, we examined 48 field sites along the Eurasian steppe eastern transect (ESET) region. In contrast to the Northeast China Transect, which includes native grassland vegetation arrayed across a regional precipitation gradient [21], the ESET region mainly reflects the changes in native grassland vegetation along a temperature gradient and under different policies [39]. We analyzed the soil organic carbon density (SOCD) in the top 30 cm and explored the relationships between the SOCD and climatic variables, soil texture, grazing intensity and community biomass productivity. We aimed to answer the following two important questions: (1) Are there significant difference in soil organic carbon storage between the Inner Mongolia grassland subregion and the Mongolia grassland subregion? (2) What are the driving factors responsible for the differences in soil organic carbon storage between these two grasslands?

## Methods

### Ethics statement

All of the survey sites were owned and/or managed by pastoral farmers, who gave permission for the survey. The field studies did not involve any endangered or protected species.

### 2.1 Study area

**Fig 1:**
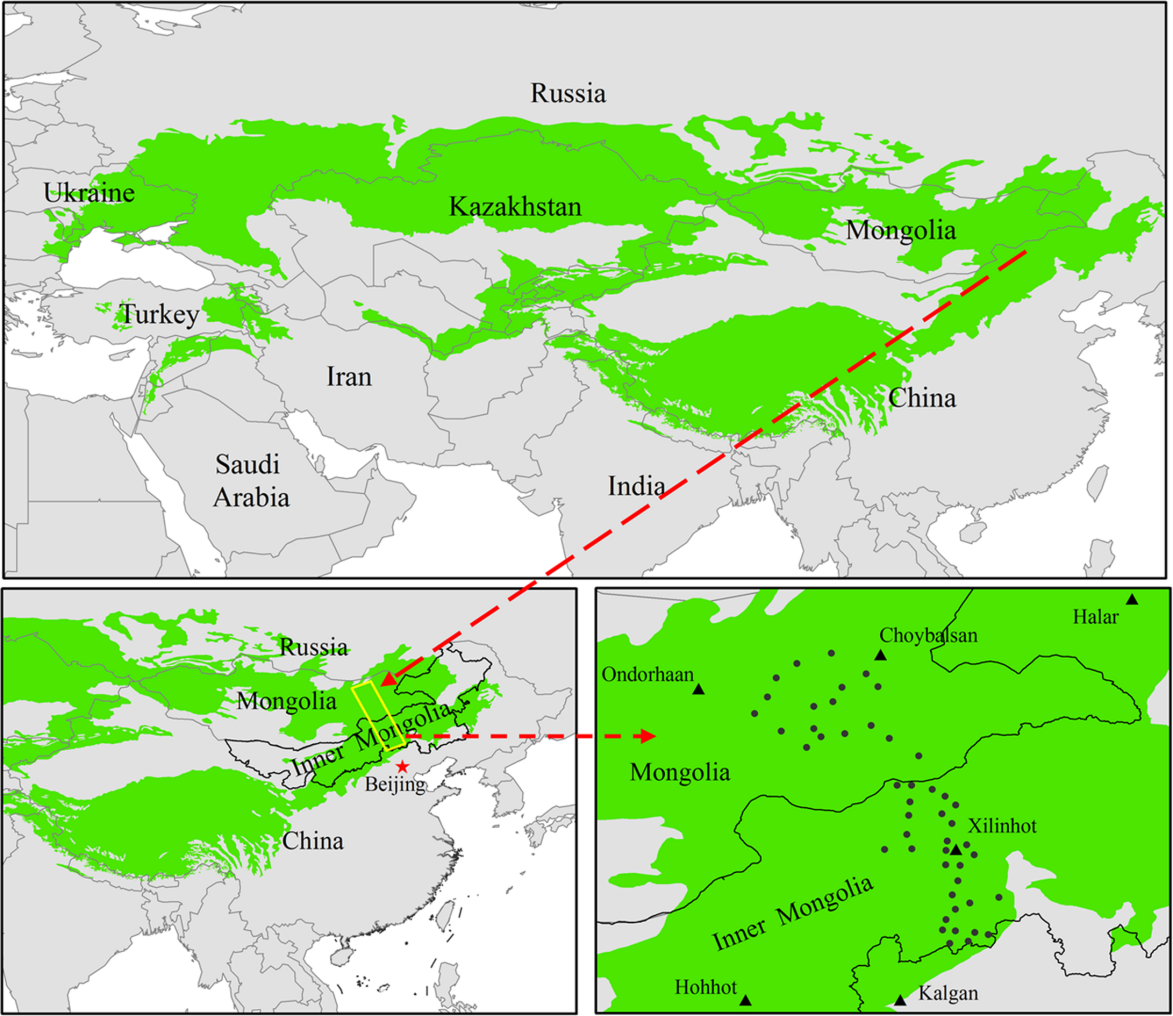
Study area and field sampling sites. Green indicates the distribution of the Eurasian steppe. The yellow transect is the study area, corresponding to the Eurasian steppe eastern transect (ESET) region. Black triangles represent the main cities in the study area. Gray circles indicate the field sampling sites.

Our study was conducted in the ESET region, which includes the Inner Mongolia grassland subregion and Mongolia grassland subregion (Fig 1). The ESET region stretches from 41.67°N to 53.56°N latitude and from 108.17°E to 115.72°E longitude (Fig 1). The mean annual temperature (MAT) ranges from -1.3 to 2.1°C, and the mean annual precipitation (MAP) varies from approximately 150 to 400 mm. The typical landforms in the ESET region are gently rolling plains, tablelands, and hills. The common soil types in the region are chernozems, chestnut soils and calcic brown soils. The vegetation types along the ESET region mainly include meadow steppe, typical steppe and desert steppe.

### 2.2 Data collection

Field sampling was performed from late July to mid-August 2012, when the grassland community biomass was at its peak. Forty-eight field sites were established from the south to the north along the ESET region, and these included 30 in the Inner Mongolia grassland subregion and 18 in the Mongolia grassland subregion (Fig 1). The position of each site was located with a GPS (S1 Table). At each site, an area of 10 × 10 m was delineated, and three 1 × 1 m quadrats were then randomly placed within this area. We harvested the aboveground parts of the plants in each quadrat, and the dry weight of the plants was obtained by oven-drying at 60°C for 24 h until a constant weight was obtained. The average aboveground biomass productivity (BP) from the three quadrats was employed as a measure of the community productivity at each site (S1 Table). Three soil profiles were collected within each quadrat. Each soil profile was extracted to a depth of 0.3 m, and the samples were divided into depth increments of 0–0.1 m, 0.1–0.2 m and 0.2–0.3 m. Each of these samples was collected using a standard container with a volume of 100 cm^3^ and a cloth pocket. All of the soil samples were air-dried and then oven-dried at 105°C to determine their bulk densities. Before the soil samples were further analyzed to determine the soil organic carbon concentration and soil texture, visible plant roots and rock fragments were manually removed by sieving the samples through a 2 mm screen [4]. The soil organic carbon concentration was measured via wet combustion with K_2_Cr_2_O_7_ [40]. A Mastersizer S3500 instrument (Microtrac Incorporated, USA) was employed to measure the soil texture, including the clay content (<0.002 mm), silt content (0.002–0.02 mm), and sand content (0.02–2 mm). The soil pH was measured using a PHS-3S pH meter (Sartorius, Germany) with a soil-water suspension (soil: water = 1:2) [21]. For each soil layer, the soil organic carbon concentration, soil bulk density and soil pH were represented by the average of the values from three spatially random profiles. The soil texture was measured using a composited sample for each soil layer from the three soil profiles.

The MAP and MAT were obtained at a 30 arc-second (~1 km^2^) resolution from the WorldClim database (www.worldclim.org) (S1 Table) [41].

Grazing is the main human activity in the region. The spatial distribution of sheep density was used as an indicator of the grazing intensity in the region. Sheep density data from the year 2012 were provided by the Food and Agriculture Organization of the United Nations (http://data.fao.org/map) and were extracted with respect to each site’s location (S1 Table).

### 2.3 Data analysis

The soil organic carbon storage was assessed using the SOCD in the three soil layers (0–0.1 m, 0.1–0.2 m, and 0.2–0.3 m) and the sum for the three layers. The SOCD at a depth of h (m) was calculated as follows:

$$SOCD_{h}=\sum_{i=1}^{n}\frac{(1-\delta_{i}\%)\times \rho_{i}\times C_{i}\times T_{i}}{100}$$

where n is the number of the soil layer; *δ*_*i*_ is the concentration of gravel larger than 2 mm in soil layer *i* (volume percentage); *ρ*_*i*_ and *C*_*i*_ are the bulk density and the soil organic carbon concentration in soil layer *i*, respectively; and *T*_*i*_ is the thickness of soil layer *i*.

All the analyses were conducted at regional scale and subregional scale. ESET region reflects regional scale, Inner Mongolia grassland subregion and Mongolia grassland subregion reflect subregional scale. For the ESET region, Inner Mongolia grassland subregion and Mongolia grassland subregion, the SOCD of each soil layer and the sum of all three soil layers are presented as the means ± standard errors. Two-way analysis of variance (ANOVA) was used to test for significant differences in the SOCD between the Inner Mongolia grassland subregion and the Mongolia grassland subregion.

We employed an ordinary least squares regression to examine the relationships between the SOCD of four soil layers (0–10 cm, 10–20 cm, 20–30 cm, and 0–30 cm) and each of the different controlling factors (MAT, MAP, clay content, soil pH, and grazing intensity) along the ESET region.

**Fig 2:**
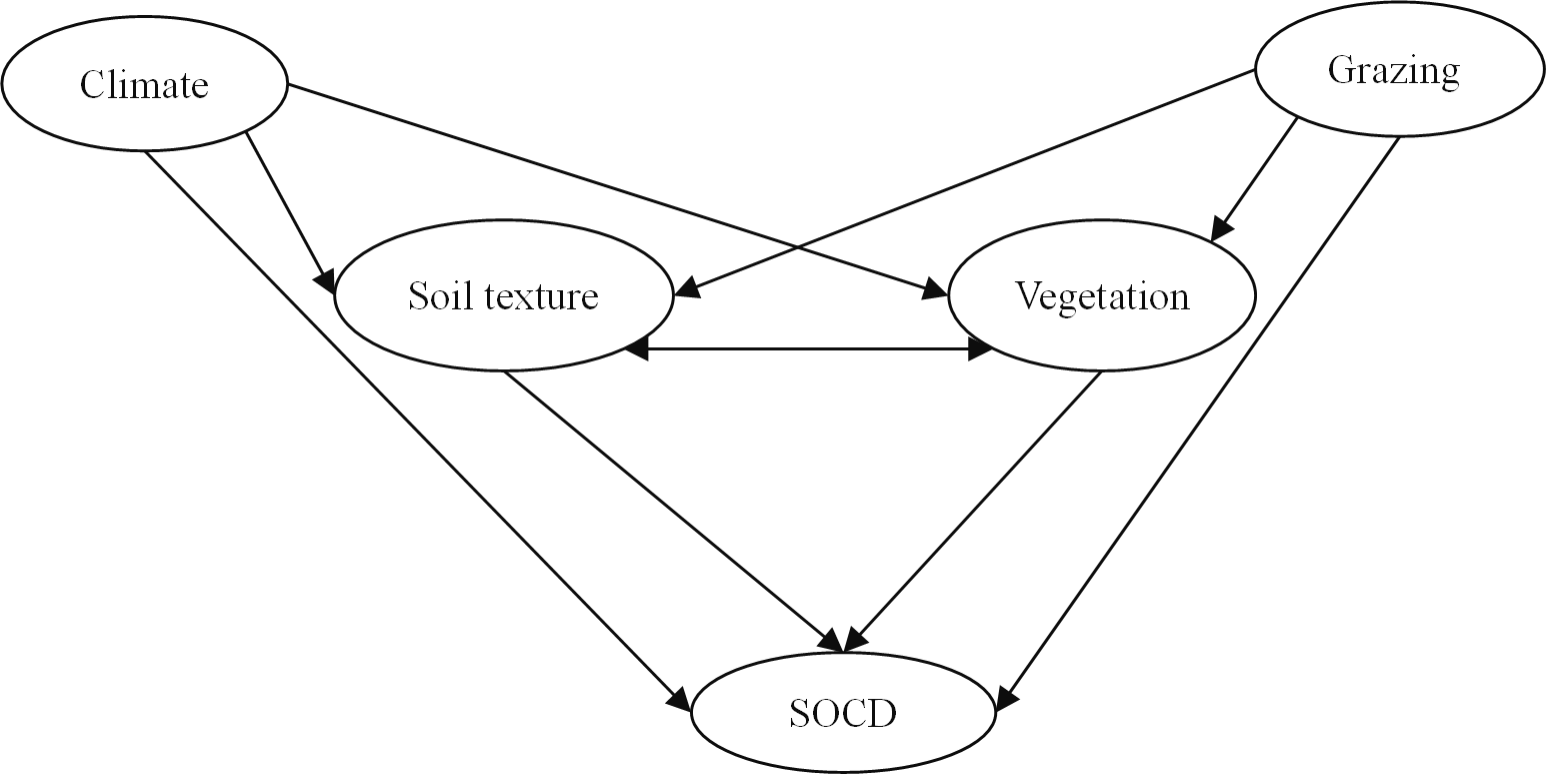
A structure equation model showing the hypothesized causal paths to examine the effects of climate, grazing, soil texture and vegetation on the soil organic carbon density (SOCD). Ellipses denote the latent variables included in the model. Single-headed arrows denote the paths. Double-headed arrows indicate an interaction effect between a pair of variables.

To analyze the relative importance of climate, soil texture, grazing and vegetation on the SOCD at 0–30 cm, we performed structural equation modeling (SEM) to examine the standardized total effect of climate, soil texture, grazing and vegetation on the SOCD on the ESET region, Inner Mongolia grassland subregion and Mongolia grassland subregion [6,17]. The climatic variables in the framework included the MAT and MAP. Because a strong linear correlation was found among the sand content, silt content and clay content, we represented the soil texture using the clay content and soil pH, and we used the grazing intensity to represent the grazing activity. Because productivity is the most comprehensive indicator of the vegetation community [42], community biomass productivity was employed to represent vegetation. In this framework, we hypothesized that climate, soil texture and grazing would directly influence the SOCD and that climate and grazing would affect the soil texture. Furthermore, climate, soil texture and grazing would indirectly influence the SOCD through community productivity. The soil texture and community productivity also showed an interaction relationship (Fig 2). A good-fitting model was indicated by a Chi-square (CMIN) analysis with a P-value > 0.05, a root-mean square error of approximation (RMSEA) < 0.05, and lower values for the Akaike information criterion (AIC) and the Browne-Cudeck criterion (BCC) [43].

Furthermore, to describe the effects of individual controlling factors (including the MAT, MAP, clay content, BP, soil pH and grazing intensity) and further identify the most important individual influences on the SOCD, a random forest regression analysis was performed to assess the importance of these factors in explaining the SOCD patterns by ranking the significance of these factors along the ESET region, Inner Mongolia grassland subregion and Mongolia grassland subregion. The significance of each factor was determined according to node purity, which was measured by the residual sum of squares, with a high node purity indicating a greater effect [44].

The SEM analysis was performed using AMOS 17.0. The random forest analysis, ANOVA and ordinary least squares regression were performed with the software package R (http://cran.r-project.org/).

## Results

### 3.1 Difference of the SOCD and controlling factors along the ESET region

**Fig 3:**
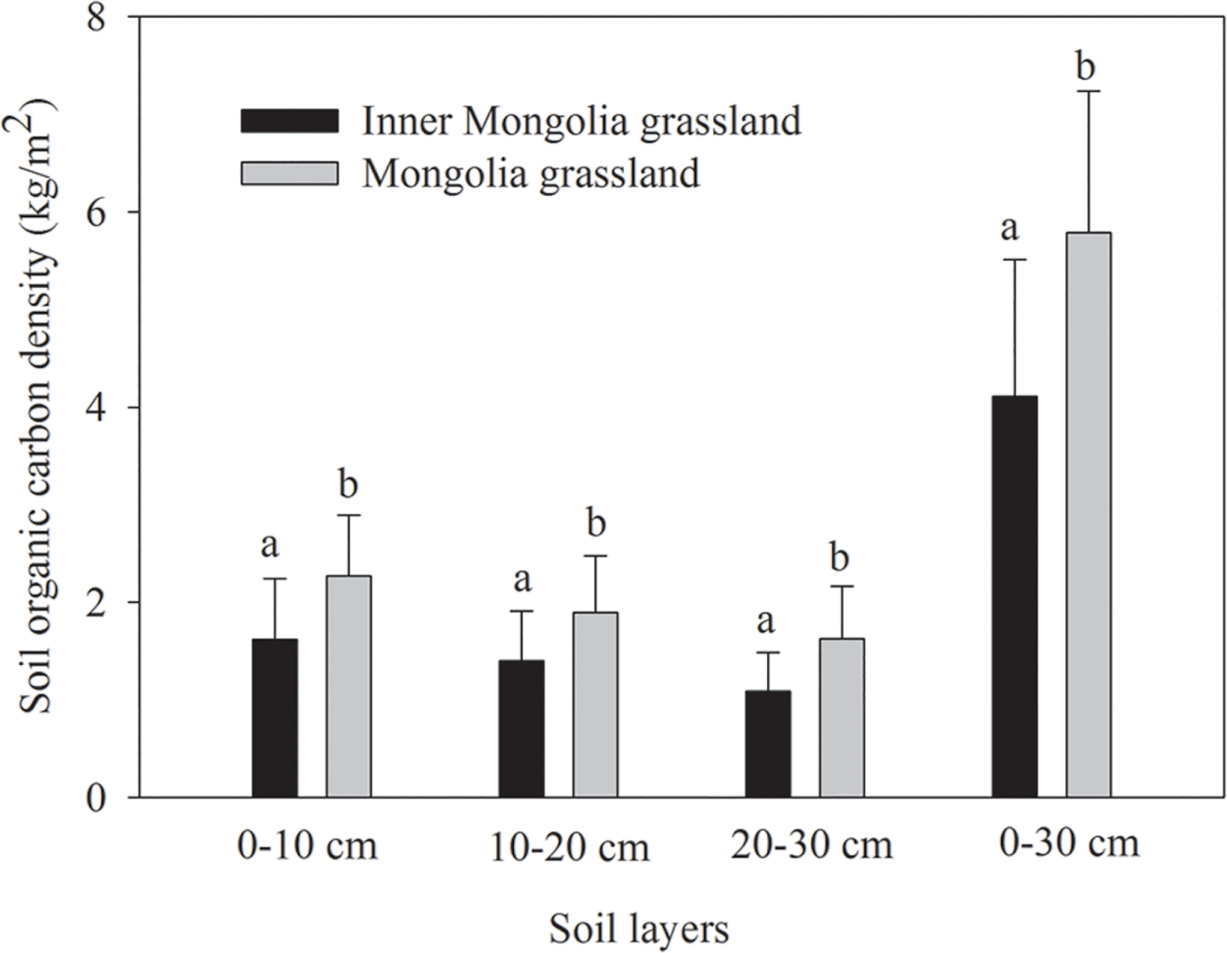
Distribution of the soil organic carbon density (SOCD) in the Inner Mongolia subregion and Mongolia grasslands subregion. Black indicates the SOCD of the Inner Mongolia grassland subregion in different soil layers. Gray indicates the SOCD of the Mongolia grassland subregion in different soil layers. Significant differences in the SOCD between the Inner Mongolia grassland subregion and the Mongolia grassland subregion are indicated by different lowercase letters.

At soil depths of 0–30 cm, the SOCD of the Inner Mongolia grassland subregion and Mongolia grassland subregion were 4.11 kg/m^2^ and 5.79 kg/m^2^, respectively (Table 1 and S1 Table). The SOCD of the Mongolia grassland subregion was significantly higher than that of the Inner Mongolia grassland subregion (Fig 3). The controlling factors also show significant difference between these two subregions. In addition to soil clay content, MAT, MAP, soil pH, biomass productivity and grazing intensity of Inner Mongolia grassland subregion were higher than that of Mongolia grassland subregion (Table 1).

**Table 1:** Environmental characteristics and soil organic carbon density (SOCD) in the 0–30 cm soil layer in the Eurasian steppe eastern transect (ESET) region, Inner Mongolia grassland (IMG) subregion and Mongolia grassland (MG) subregion.

Area	SOCD (kg/m^2^)	MAT (°C)	MAP (mm)	SCC (%)	Soil pH	BP (g/m^2^)	GI (sheep/km^2^)
EETS	4.74±1.63	0.99±0.77	270.83±65.76	28.96±18.54	7.51±0.60	225.46±79.93	44.64±34.12
IMG	4.11±1.40	1.22±0.82	308.10±50.57	20.60±12.63	7.75±0.26	249.73±57.29	65.88±25.03
MG	5.79±1.45	0.62±0.50	208.72±32.51	42.89±18.71	7.10±0.77	185.02±96.40	9.23±5.18

Note: MAT, mean annual temperature; MAP, mean annual precipitation; SCC, soil clay content; BP, community biomass productivity; GI, grazing intensity.

### 3.2 Vertical distribution of the SOCD along the ESET region

For the three soil layers of 0–10 cm, 10–20 cm and 20–30 cm, the SOCD of the Inner Mongolia grassland subregion were 1.62 kg/m^2^, 1.40 kg/m^2^ and 1.10 kg/m^2^, and the SOCD of the Mongolia grassland subregion were 2.27 kg/m^2^, 1.89 kg/m^2^ and 1.63 kg/m^2^, respectively (Fig 3). All three areas showed decreases in the SOCD with increasing soil depth, and the highest SOCD was detected in the 0–10 cm layer. Meanwhile, for each soil layer, the SOCD showed significant negative relationships with the MAT, MAP and grazing intensity along the ESET region (Table 2).

**Table 2:** Relationships between the soil organic carbon density (SOCD) and a series of controlling factors in three soil layers along the ESET region.

Soil layer	MAT	MAP	Soil pH	BP	GI
SOCD 0–10 cm	-0.494**	-0.356**	-0.023	-0.095	-0.436**
SOCD 10–20 cm	-0.483**	-0.304*	0.078	-0.222	-0.434**
SOCD 20–30 cm	-0.456**	-0.382**	-0.152	-0.274	-0.466**
SOCD 0–30 cm	-.531**	-.376**	-0.03	-0.208	-0.492**

Note: MAT, mean annual temperature; MAP, mean annual precipitation; BP, community biomass productivity; GI, grazing intensity. A single asterisk indicates P<0.05, and double asterisks indicate P<0.01.

### 3.3 Effects of climate, soil texture, grazing and vegetation on the SOCD

**Fig 4:**
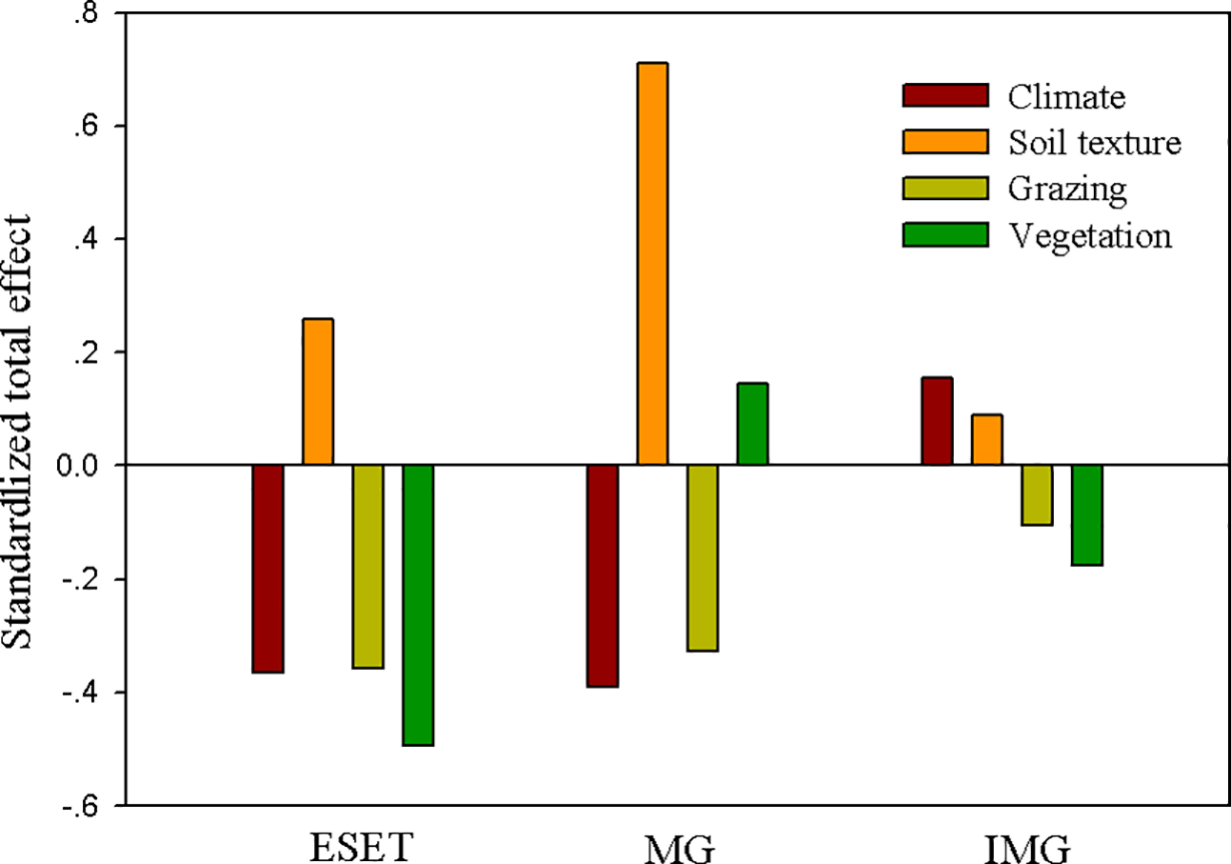
Standardized total effect (direct plus indirect) of climate, soil texture, grazing and vegetation on the soil organic carbon density (SOCD). The standardized total effect of each variable is shown separately with different colors. ESET, Eurasian steppe eastern transect; MG, Mongolia grassland; IMG, Inner Mongolia grassland.

**Fig 5:**
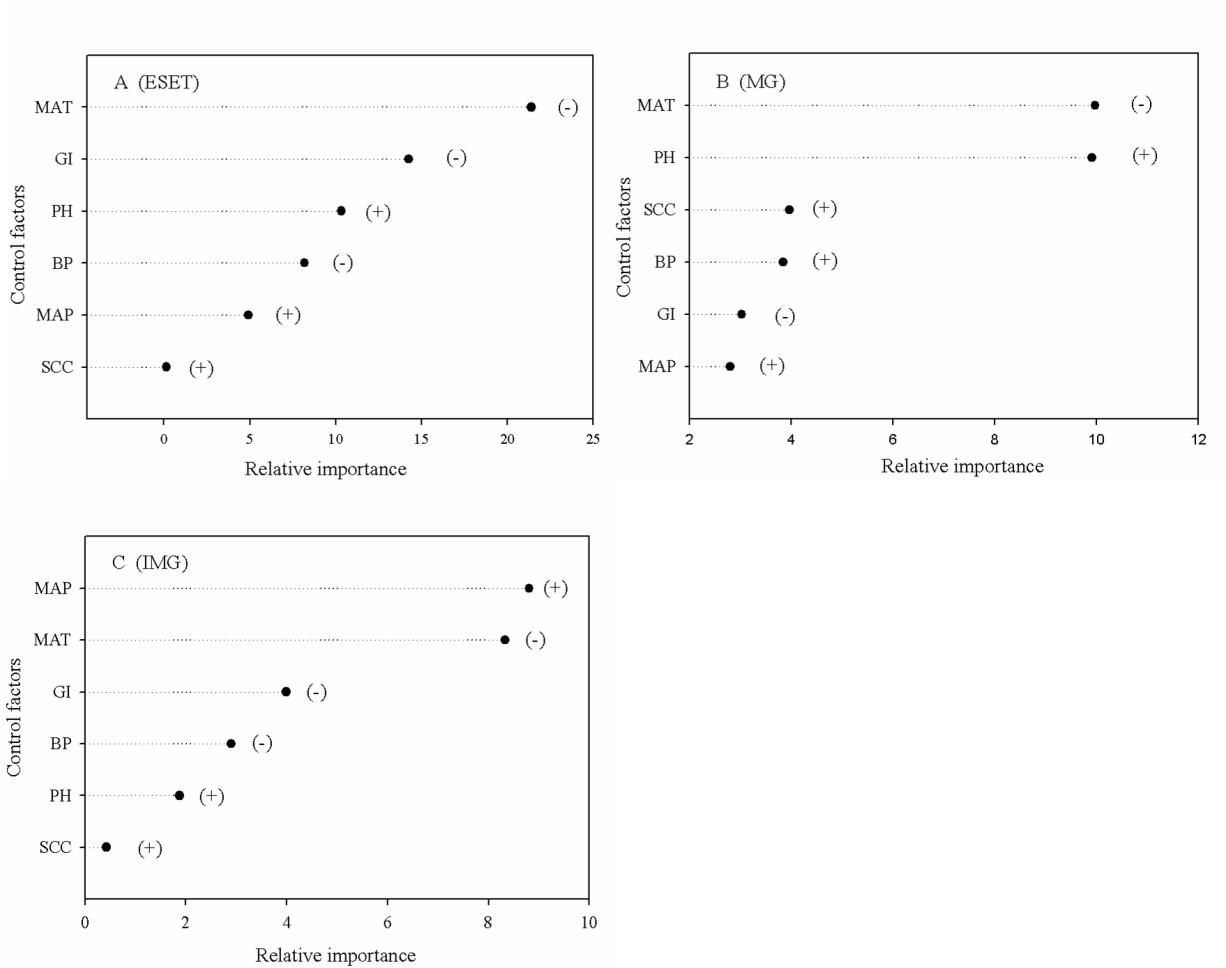
Relative importance of individual factors in controlling the soil organic carbon density in the ESET region (A), Mongolia grassland subregion (B) and Inner Mongolia grassland subregion (C). ESET, Eurasian steppe eastern transect; MG, Mongolia grassland; IMG, Inner Mongolia grassland; MAP, mean annual precipitation; MAT, mean annual temperature; GI, grazing intensity; SCC, soil clay content; PH, soil pH; BP, biomass productivity. (+) and (-) indicate positive and negative effects of the controlling factor.

In the ESET region, vegetation, climate and grazing played a crucial role in determining the SOCD (Fig 4). MAT and grazing intensity were the two most important factors controlling the SOCD, and both factors had a negative effect (Fig 5A). In the Mongolia grassland subregion, soil texture and climate played a key role in determining the SOCD (Fig 4). The MAT and soil pH were the two most important factors controlling the SOCD, and these factors exerted a negative effect and a positive effect, respectively (Fig 5B). In the Inner Mongolia grassland subregion, climate represented the dominant factor determining the SOCD (Fig 4). The MAP and MAT were the most important factors controlling the SOCD, with the former having a positive effect and the latter having a negative effect (Fig 5C).

## Discussion

### 4.1 SOCD estimates and controlling factors along the ESET region

Estimations of soil organic carbon storage are vital for quantifying the soil carbon sequestration potential and addressing global climate change [2,4]. In this study, we found that the SOCD at a depth of 0–30 cm along the ESET region was 4.74 kg/m^2^ (Table 1 and S1 Table). This value was higher than the SOCD values determined in the 0–30 cm layer in grasslands in other parts of the world, such as the Indian tropical grassland (3.8 kg/m^2^) [45], Nigerian savannah (4.2 kg/m^2^) [46], and Brazilian savannah (3.4 kg/m^2^) [47]. However, the SOCD of the ESET region at 0–30 cm was lower than that of the alpine grassland of the Qinghai-Tibet Plateau (7.4 kg/m^2^) [48], Sanjiang plain grassland (10.65 kg/m^2^) [4], Belgian grassland (9.22 kg/m^2^) [8] and Irish grassland (11.1 kg/m^2^) [49]. Moreover, the SOCD of the ESET region at 0–30 cm was similar to that of the Australian temperate grassland (4.9 kg/m^2^) [6], New Zealand grassland (6.83 kg/m^2^) [50], Iranian grassland (6.33 kg/m^2^)[51] and American temperate grassland (5.5 kg/m^2^) [52]. These values are consistent with the global grassland soil organic carbon storage patterns derived from model simulations [53,54], which show that the soil organic carbon storage of tropical savannah is lower than that of temperate grasslands and that colder grasslands exhibit higher soil organic carbon storage (Table 3).

**Table 3:** Soil organic carbon density over 0–30 cm and mean annual precipitation (MAP) and temperature (MAT) in various grasslands.

Climatic zone	Grassland type	Country/Region	MAP (mm)	MAT (°C)	SOCD (kg/m^2^)
Tropic	African savanna	Nigeria	1500	29	4.20
	South American tropical grassland	Brazil	2200	25.6	3.40
	Indian tropical grassland	New Delhi, India	650	28	3.80
Temperate	Australian temperate grassland	Eastern Australia	1060	16	4.90
	New Zealand temperate grassland	New Zealand	1900	18	6.83
	North American prairie	Ohio, America	10.7	953	5.50
	Eurasian steppe	Inner Mongolia	308	1.22	4.11
	Eurasian steppe	Mongolia	209	0.62	5.79
	Eurasian steppe	Lorestan province, Iran	450	12	6.33
	Eurasian steppe	Three Rivers Source Region of the Tibetan Plateau	417	-0.64	7.40
	Eurasian steppe	Sanjiang plain of China	575	2.85	10.65
	Eurasian steppe	Belgium	1000	9	9.22
	Eurasian steppe	Ireland	900	9.7	11.10

A number of studies have demonstrated that soil organic carbon storage decreases with increasing soil depth [7,13], and our results support this view (Fig 3). Jobbagy and Jackson [13] found that a vertical distribution pattern of soil organic carbon storage is strongly correlated with the vegetation type. Our study was conducted in grasslands, where the soil organic carbon input is mainly derived from the decomposition of aboveground litter and underground roots. Organic matter derived from the decomposition of aboveground litter will be transferred to shallow soil and accumulate in the 0–10 cm soil layer, whereas plant roots are concentrated at a soil depth of 0–20 cm along the ESET region. Thus, the highest SOCD was observed in the 0–10 cm soil layer. Some other studies have demonstrated that soil organic carbon storage in topsoil is mainly affected by climate, whereas that in subsoil is mainly controlled by soil texture [3,17]. However, we found that the relationships between the SOCD and a series of controlling factors were the same among the three soil layers (Table 2). This finding can mainly be explained by the fact that these three soil layers (0–10 cm, 10–20 cm, and 20–30 cm) all belong to topsoil, and soil at a depth greater than 30 cm is considered subsoil [17].

The SEM analysis showed that climate and grazing play the most important roles in determining the SOCD along the ESET region. Based on the random forest regression analysis, we found that the MAT was the most crucial climate factor, and the SOCD decreased with increasing MAT (Fig 5A). This result is consistent with the results from previous studies conducted in the US Great Plains [12], France [55], Germany [11] and globally [13]. With an increase in temperature, evaporation is enhanced, and plant productivity is reduced, ultimately resulting in a decrease in soil organic carbon input [11,55]. In contrast, soil microbial decomposition activity is enhanced with increasing temperature, which leads to a higher soil organic carbon output [4]. Meanwhile, we observed that grazing was the second most important factor controlling soil organic carbon storage, and the SOCD decreased gradually with increasing grazing intensity (Fig 5A and Table 2). Our findings support many previous studies which shown that a higher grazing intensity results in reduced soil organic carbon storage [31]. Grazing can directly inhibit the soil carbon input by reducing plant productivity and litter [56]. In contrast, grazing reduces vegetation coverage, which aggravates soil erosion and reduces the soil clay content. A decrease in the soil clay content is not conducive to the fixation of soil organic matter and indirectly enhances the decomposition of soil organic carbon, ultimately reducing the soil organic carbon content [22].

### 4.2 Controlling factors of the SOCD in the Inner Mongolia grassland subregion and Mongolia grassland subregion

**Fig 6:**
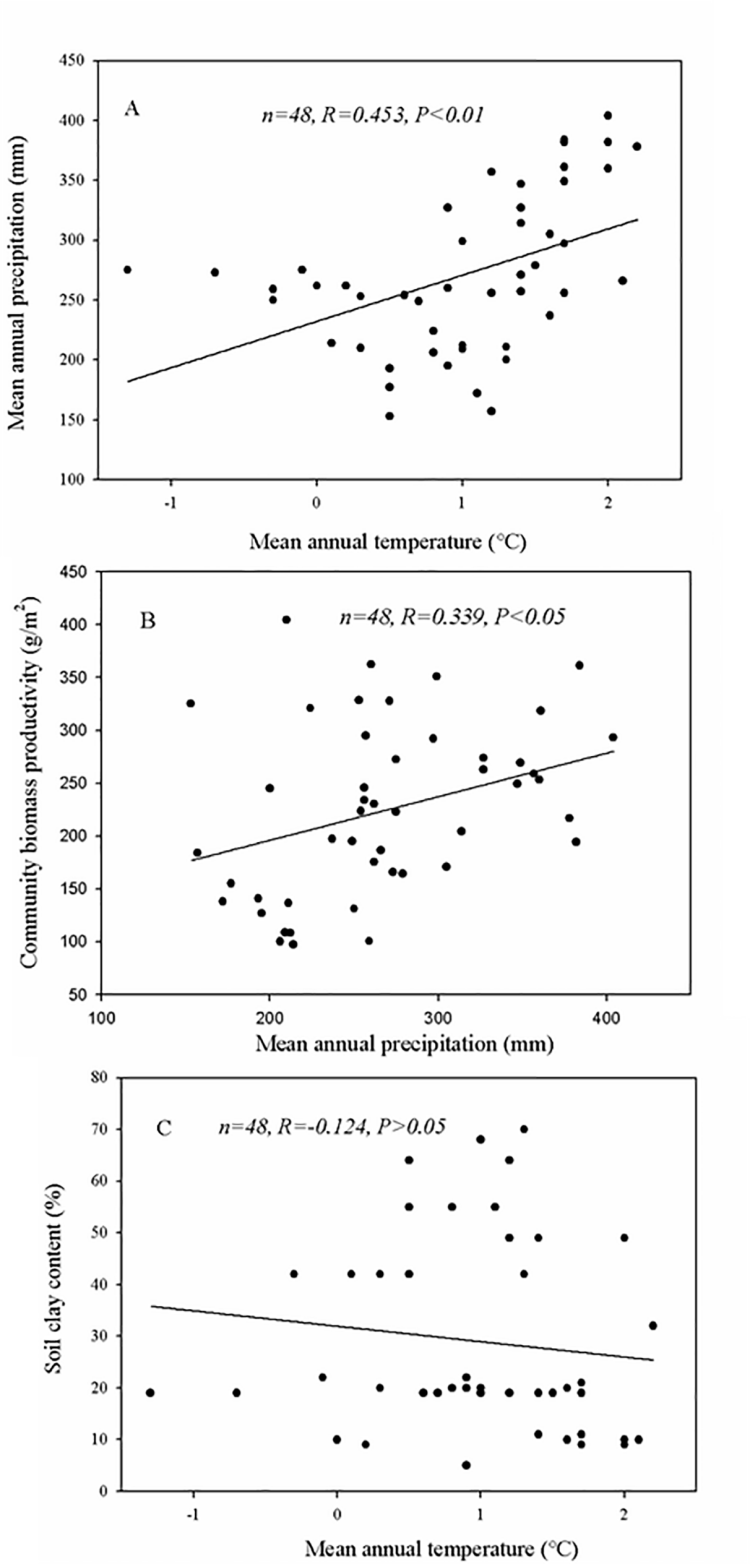
(A) Significant positive linear relationship between the mean annual temperature and mean annual precipitation along the ESET region. (B) Significant positive linear relationship between the mean annual precipitation and community biomass productivity along the ESET region. (C) Non-significant negative linear relationship between the mean annual temperature and soil clay content along the ESET region. ESET, Eurasian steppe eastern transect.

The MAP was identified as the most important factor controlling soil organic carbon in the Inner Mongolia grassland subregion (Fig 5C). This result is consistent with those of He *et al*. [21]. Precipitation has been shown to be the most important limiting factor of Inner Mongolia grassland subregion productivity [57,58]. However, our results showed a negative relationship between the MAP, BP and soil organic carbon storage along the ESET region (Table 2). We hypothesize that this unusual result was due to the colinearity of precipitation and temperature along the ESET region, where a significant positive linear relationship between the MAT and MAP was observed (Fig 6A). Although, increasing MAP leads to an increase in biomass productivity (Fig 6B), simultaneous increases in the MAT and MAP reduce the efficiency of MAP alone in increasing biomass productivity in arid climates. We also found that the soil clay content (SCC) decreased with increasing MAT (Fig 6C). A low SCC is more conducive to the decomposition of soil organic carbon [19,21]. Considering MAT is the dominant controlling factor on the SOCD and exerts a negative effect (Fig 5A), the effect of MAP and biomass productivity on SOCD is significantly regulated by MAT. Therefore, a negative relationship was observed between the MAP, biomass productivity and soil organic carbon storage along the ESET region.

Consistent with the results along the ESET region, the MAT was determined to be the most important factor in soil organic carbon storage in the Mongolia grassland subregion (Fig 5B). Moreover, the soil pH was the second most important factor controlling soil carbon storage in the Mongolia grassland subregion (Fig 5B). Many studies have shown that the soil pH is closely related to soil organic carbon storage [9,15]. The soil pH mainly affects soil microbial activity, which directly affects the decomposition of soil organic matter [59]. Both large-scale field investigations [60] and long-term controlled experiments [20] have found that the total abundance of soil microorganisms is reduced with an increasing soil pH in the Mongolia grassland subregion. A decrease in the soil microbial content indicates that a decrease in microbial activity will occur, leading to reduced soil microbial decomposition of soil organic matter and ultimately resulting in an increase in soil organic carbon. Thus, an increase in the soil pH results in the accumulation of soil organic carbon.

Both the Inner Mongolia grassland subregion and the Mongolia grassland subregion belong to the eastern Eurasian steppe, and these two grasslands exhibit highly similar vegetation types [35]. However, our study showed that the crucial factors controlling soil organic carbon storage were widely divergent between these two regions (Fig 5). There might be two main reasons for this difference. First, temperature and precipitation play different roles in plant productivity, which further determines the carbon input. The relative importance of precipitation is more prominent in the Inner Mongolia grassland subregion[20,58]. In contrast, with a decrease in temperature, the relative importance of temperature for plant productivity is enhanced [57,58]. We found that the MAT of the Mongolia grassland subregion (0.62°C) was markedly lower than that of the Inner Mongolia grassland subregion (1.22°C) (Table 1 and S1 Table). Thus, the relative importance of temperature in determining plant productivity in the Mongolia grassland subregion is greater than that in the Inner Mongolia grassland subregion. Second, there is a significant difference in grazing intensity between the Inner Mongolia grassland subregion and the Mongolia grassland subregion. Over the years, the Inner Mongolia grassland subregion has been subject to a high grazing intensity [35]. This continuous high grazing intensity has resulted in a greatly decreased SCC (only 20.60%) and an increased soil pH (approximately 7.75) (Table 1 and S1 Table). A low soil clay content and a high soil pH inevitably reduce the regulatory ability of soil texture on soil organic carbon [21]. Thus, the soil texture does not have a significant effect on soil organic carbon storage in the Inner Mongolia grassland subregion. Compared with the grazing intensity in the Inner Mongolia grassland subregion (approximately 65.88 sheep/hm^2^), the grazing intensity in the Mongolia grassland subregion is markedly lower (only 9.23 sheep/hm^2^) (Table 1 and S1 Table), and the soil is in a relatively primitive state and shows high spatial heterogeneity. Compared with the soil clay content and soil pH, soil organic carbon storage is more sensitive to soil pH [9,15]. Thus, soil pH is a crucial factor controlling soil organic carbon storage in the Mongolia grassland subregion. In summary, temperature and grazing intensity drive the differences in soil organic carbon storage between the Inner Mongolia grassland subregion and the Mongolia grassland subregion along the ESET region.

## Supporting information

S1 Table Location, environmental characteristics, community biomass productivity and soil carbon storage of 48 field sites on the Eurasian steppe eastern transect. MAP, mean annual precipitation; MAT, mean annual temperature; SCC, soil clay content; GI, grazing intensity; BP, biomass productivity; SOCD, soil organic carbon density.
(DOCX)
