# Supplementary material for: Effects of temperature and grazing on soil organic carbon storage in grasslands along the Eurasian steppe eastern transect
Source: PLoS One. 2017 Oct 30;12(10):e0186980. doi: 10.1371/journal.pone.0186980 (PMC5662174; doi:10.1371/journal.pone.0186980)
Supplement: S1 Table — MAP, mean annual precipitation; MAT, mean annual temperature; SCC, soil clay content; GI, grazing intensity; BP, biomass productivity; SOCD, soil organic carbon density. (DOCX) [file pone.0186980.s001.docx]

**Supporting information**

S1 Table. **Location, environmental characteristics, community biomass productivity and soil carbon storage of 48 field sites on the Eurasian steppe eastern transect.** MAP, mean annual precipitation; MAT, mean annual temperature; SCC, soil clay content; GI, grazing intensity; BP, biomass productivity; SOCD, soil organic carbon density.

| Site code | Longtitude(°) | Lattitude(°) | Administrative  region | MAP(mm) | MAT(℃) | SCC(%) | Soil pH | GI(sheep/km^2^) | BP(g/m^2^) | SOCD of 0-30 cm  (kg/m^2^) |
| --- | --- | --- | --- | --- | --- | --- | --- | --- | --- | --- |
| 1 | 115.94 | 42.00 | Inner Mongolia | 382.00 | 2.00 | 9.00 | 7.69 | 58.30 | 194.62 | 55.61 |
| 2 | 116.33 | 42.05 | Inner Mongolia | 384.00 | 1.70 | 11.00 | 7.53 | 126.13 | 361.48 | 30.22 |
| 3 | 116.74 | 42.18 | Inner Mongolia | 404.00 | 2.00 | 49.00 | 7.57 | 127.28 | 293.24 | 22.28 |
| 4 | 116.46 | 42.23 | Inner Mongolia | 378.00 | 2.20 | 32.00 | 7.53 | 117.83 | 217.06 | 26.96 |
| 5 | 116.06 | 42.27 | Inner Mongolia | 360.00 | 2.00 | 10.00 | 7.71 | 26.96 | 253.4 | 37.04 |
| 6 | 115.79 | 42.28 | Inner Mongolia | 361.00 | 1.70 | 19.00 | 7.76 | 78.64 | 318.54 | 49.16 |
| 7 | 115.86 | 42.50 | Inner Mongolia | 349.00 | 1.70 | 9.00 | 7.77 | 53.60 | 269.42 | 37.39 |
| 8 | 116.06 | 42.72 | Inner Mongolia | 347.00 | 1.40 | 49.00 | 8.01 | 97.57 | 249.26 | 52.87 |
| 9 | 116.35 | 42.85 | Inner Mongolia | 357.00 | 1.20 | 49.00 | 7.73 | 76.86 | 259.08 | 40.97 |
| 10 | 116.97 | 42.97 | Inner Mongolia | 382.00 | 1.70 | 21.00 | 7.70 | 39.18 | 194.08 | 27.51 |
| 11 | 115.99 | 43.02 | Inner Mongolia | 327.00 | 1.40 | 49.00 | 7.68 | 78.27 | 263.12 | 31.75 |
| 12 | 116.11 | 43.32 | Inner Mongolia | 327.00 | 0.90 | 22.00 | 7.91 | 76.77 | 273.88 | 48.07 |
| 13 | 116.15 | 43.63 | Inner Mongolia | 305.00 | 1.60 | 20.00 | 8.00 | 61.11 | 171.18 | 30.49 |
| 14 | 115.85 | 43.65 | Inner Mongolia | 299.00 | 1.00 | 19.00 | 8.34 | 58.96 | 351.18 | 75.48 |
| 15 | 116.45 | 43.86 | Inner Mongolia | 314.00 | 1.40 | 11.00 | 7.69 | 84.23 | 204.46 | 54.03 |
| 16 | 115.86 | 43.95 | Inner Mongolia | 279.00 | 1.50 | 19.00 | 7.79 | 55.53 | 164.78 | 52.99 |
| 17 | 114.57 | 43.97 | Inner Mongolia | 237.00 | 1.60 | 10.00 | 7.73 | 66.39 | 197.22 | 26.35 |
| 18 | 115.14 | 43.98 | Inner Mongolia | 260.00 | 0.90 | 5.00 | 7.52 | 47.41 | 362.4 | 40.88 |
| 19 | 116.29 | 44.07 | Inner Mongolia | 297.00 | 1.70 | 11.00 | 8.15 | 62.98 | 292.38 | 42.67 |
| 20 | 115.89 | 44.15 | Inner Mongolia | 266.00 | 2.10 | 10.00 | 8.15 | 43.01 | 186.7 | 35.63 |
| 21 | 115.04 | 44.29 | Inner Mongolia | 254.00 | 0.60 | 19.00 | 7.42 | 49.13 | 223.84 | 33.04 |
| 22 | 115.98 | 44.52 | Inner Mongolia | 271.00 | 1.40 | 19.00 | 8.26 | 45.61 | 327.9 | 35.83 |
| 23 | 115.08 | 44.68 | Inner Mongolia | 249.00 | 0.70 | 19.00 | 7.69 | 60.81 | 195.08 | 40.95 |
| 24 | 115.78 | 44.72 | Inner Mongolia | 257.00 | 1.40 | 19.00 | 7.87 | 41.38 | 295.16 | 32.26 |
| 25 | 116.06 | 44.90 | Inner Mongolia | 256.00 | 1.70 | 19.00 | 7.32 | 75.89 | 234.22 | 23.94 |
| 26 | 115.10 | 44.97 | Inner Mongolia | 262.00 | 0.00 | 10.00 | 7.84 | 48.05 | 230.52 | 23.30 |
| 27 | 115.84 | 45.08 | Inner Mongolia | 256.00 | 1.20 | 19.00 | 7.25 | 63.52 | 245.76 | 34.51 |
| 28 | 115.57 | 45.23 | Inner Mongolia | 275.00 | -0.10 | 22.00 | 7.90 | 65.03 | 272.54 | 66.68 |
| 29 | 115.14 | 45.32 | Inner Mongolia | 273.00 | -0.70 | 19.00 | 7.58 | 50.87 | 166.16 | 68.34 |
| 30 | 114.84 | 45.32 | Inner Mongolia | 275.00 | -1.30 | 19.00 | 7.47 | 39.12 | 223.26 | 55.14 |
| 31 | 115.29 | 45.93 | Mongolia | 262.00 | 0.20 | 9.00 | 8.40 | 5.56 | 175.55 | 79.54 |
| 32 | 112.94 | 46.11 | Mongolia | 209.00 | 1.00 | 68.00 | 8.17 | 16.65 | 108.88 | 61.96 |
| 33 | 114.67 | 46.30 | Mongolia | 212.00 | 1.00 | 20.00 | 7.60 | 2.63 | 108.63 | 69.47 |
| 34 | 113.24 | 46.33 | Mongolia | 206.00 | 0.80 | 20.00 | 7.66 | 11.34 | 100.25 | 70.86 |
| 35 | 113.74 | 46.40 | Mongolia | 210.00 | 0.30 | 20.00 | 6.91 | 6.99 | 404.6 | 59.21 |
| 36 | 112.41 | 46.45 | Mongolia | 211.00 | 1.30 | 70.00 | 7.13 | 14.18 | 136.75 | 41.09 |
| 37 | 113.09 | 46.50 | Mongolia | 200.00 | 1.30 | 42.00 | 6.88 | 12.68 | 245.23 | 29.25 |
| 38 | 114.29 | 46.57 | Mongolia | 195.00 | 0.90 | 20.00 | 7.30 | 6.74 | 127.07 | 49.37 |
| 39 | 111.85 | 46.82 | Mongolia | 250.00 | -0.30 | 42.00 | 7.20 | 18.31 | 131.34 | 37.86 |
| 40 | 113.09 | 46.97 | Mongolia | 214.00 | 0.10 | 42.00 | 6.01 | 2.87 | 97.69 | 59.99 |
| 41 | 113.49 | 47.07 | Mongolia | 193.00 | 0.50 | 42.00 | 6.09 | 5.69 | 140.93 | 50.26 |
| 42 | 112.12 | 47.17 | Mongolia | 259.00 | -0.30 | 42.00 | 6.17 | 15.97 | 100.63 | 72.54 |
| 43 | 113.68 | 47.37 | Mongolia | 177.00 | 0.50 | 55.00 | 8.56 | 4.96 | 155.26 | 76.55 |
| 44 | 114.44 | 47.38 | Mongolia | 157.00 | 1.20 | 64.00 | 6.82 | 3.34 | 184.2 | 39.80 |
| 45 | 112.31 | 47.57 | Mongolia | 253.00 | 0.30 | 42.00 | 7.17 | 15.33 | 328.68 | 64.30 |
| 46 | 114.19 | 47.65 | Mongolia | 153.00 | 0.50 | 64.00 | 7.06 | 4.65 | 325.42 | 70.98 |
| 47 | 112.74 | 47.87 | Mongolia | 224.00 | 0.80 | 55.00 | 6.31 | 9.25 | 321.11 | 51.73 |
| 48 | 113.46 | 48.08 | Mongolia | 172.00 | 1.10 | 55.00 | 6.35 | 8.99 | 138.07 | 57.29 |
